# Prevalence of poor self-care practice and associated factors among adult asthmatic patients in Africa: systematic review and meta-analysis

**DOI:** 10.1038/s41598-026-55252-y

**Published:** 2026-05-25

**Authors:** Menberu Gete, Anteneh Lamesgen, Haile Amha, Asmamaw Getnet, Getnet Gedif, Aysheshim Asnake Abneh, Atsede Alle Ewunetie, Tadele Derbew Kassie, Abebaw Abeje Muluneh, Getachew Tilaye Mihiret, Yilkal Dagnaw Melesse, Melkamu Siferih, Alehegn Aderaw Alamneh

**Affiliations:** 1 Department of Nursing, College of Health Sciences, Debre Markos University, Debre Markos, Ethiopia; 2 Department of Public Health, College of Medicine and Health Sciences, Debre Markos University, Debre Markos, Ethiopia; 3 Department of Midwifery, College of Medicine and Health Science, Debre Markos University, Debre Markos, Ethiopia; 4 Department of Obstetrics and Gynecology, College of Medicine and Health Science, Debre Markos University, Debre Markos, Ethiopia; 5 Department of Human Nutrition, College of Medicine and Health Sciences, Debre Markos University, Debre Markos, Ethiopia

**Keywords:** Asthma, Self-care, Adult, Meta-analysis, Systematic review, Africa

## Abstract

Poor self-care practice among adult asthmatic patients is a prevalent and serious clinical concern, as it is directly associated with significantly greater morbidity and mortality. It leads to adverse outcomes, including more frequent exacerbations, increased utilization of rescue inhalers and oral steroids, higher rates of emergency department visits, quantifiable reductions in lung function, and an overall decline in quality of life. Although several primary studies in Africa have reported highly variable prevalence rates of poor self-care practice among adult populations, no study has yet synthesized these findings. Therefore, this systematic review and meta-analysis were undertaken to estimate the pooled prevalence of poor self-care practice among adult asthmatics in Africa and to identify the key associated factors. This review followed the Preferred Reporting Items for Systematic Reviews and Meta-analyses 2020 guidelines. Studies conducted among adult asthmatic patients in Africa that reported asthma self-care practice levels and/or associated factors were included, while interventional studies, reviews, articles without full-text availability, or those of low methodological quality were excluded. Data were retrieved from Web of Science, CINHAL, PubMed/MEDLINE, ScienceDirect, Google Scholar, and University repositories. The quality of the studies was assessed using the Newcastle–Ottawa Scale. A random-effects model was applied to estimate the pooled prevalence of poor self-care practice. Publication bias was assessed using Egger’s test and funnel plots, and sensitivity analysis examined the impact of individual studies on the pooled estimate. Data were extracted in Microsoft Excel, and analyses were conducted in STATA 17, with significance set at *p* < 0.05 and a 95% confidence interval. A total of eight studies with 1779 adult asthmatics were included in this systematic review and meta-analysis. The pooled prevalence of poor self-care practice was 55.47% (95% CI = 46.10, 64.84) with 95% prediction interval (26.38%, 84.55%). Several socio-demographic, clinical, behavioral, and psychosocial factors were consistently associated with poor asthma self-care practice. Interventions to improve asthma self-care practice in Africa should adopt a holistic approach that targets high-risk groups (older adults, those with low education or comorbidities), addresses modifiable behaviors such as smoking and alcohol use, supports mental health, and strengthens social support networks.

**Supplementary Information:**

The online version contains supplementary material available at 10.1038/s41598-026-55252-y.

## Introduction

Asthma is a chronic lung disease characterized by reversible airway obstruction, typically marked by chronic airway inflammation, variable symptoms including wheezing, shortness of breath (SOB), chest tightness, and/or cough, as well as variable expiratory airflow limitation. It is primarily associated with airway hyperresponsiveness to direct or indirect stimuli, and this feature may persist even after symptoms are absent or lung function returns to normal, but can be corrected with treatment^1^. If left untreated, it causes severe respiratory failure^2^.

Asthma is a serious global public health problem that affects an estimated 262 million (15.7%) people and causes 461,000 (5.4%) deaths in 2019. It is responsible for 21.6 million (5.6%) disability-adjusted life-years (DALYs), 10.2 million (15.4%) years lived with disability (YLDs) and 11.4 million (1.9%) years of life lost (YLLs) in the same year^3^. In Africa, asthma affects an estimated 51.38 million individuals (3.67%), with 25.6% of these cases classified as severe asthma^4,5^. As indicated by evidences, the best strategy to manage asthma non-pharmacologically is self-care practice (SCP)^6^. It is defined as the actions individuals take to maintain and enhance their health, prevent illness, and manage existing health conditions. It encompasses a wide range of activities, including personal hygiene, nutrition, exercise, and mental well-being practices^7^. Asthma SCP specifically refers to the proactive measures that individuals with asthma take to manage their condition effectively, including monitoring symptoms, adhering to medication regimens, identifying and avoiding triggers, and making lifestyle adjustments to minimize the risk of asthma attacks^8^.

Poor SCP among these people is prevalent and worthy of concern, as evidenced by multiple studies showing inappropriate inhaler use, failure to differentiate reliever from controller medications^9^, lack of regular physical exercise, inconsistent healthcare facility visits, staying home during asthma attacks, and persistent smoking habits^10^. These patients also neglect prescribed environmental control measures, such as proper cleaning to reduce trigger exposures^11^.

Poor SCP contribute to greater asthma morbidity and mortality^12^. Individuals with asthma who engage in poor SCP experience frequent attacks, resulting in missed school or work days, increased visits to emergency departments and urgent care, and poor health^13^. They also experience a higher frequency of oral steroid use^14^, lower quality of life scores, increased use of rescue inhalers, reduced lung function, and admissions to intensive care units^15^.

When patients do not adhere to medication guidelines, fail to recognize and avoid triggers, or neglect to monitor their symptoms effectively due to lack of knowledge, they are more likely to experience uncontrolled asthma^16^, resulting in impairments across a number of important health outcomes and represents a significant unmet need and resource burden^17^.

Different Factors are responsible for poor SCP among individuals with asthma, including advancing in age, being an alcohol drinker, poor social support, having depression, having anxiety^18,19^, knowledge deficiency, limited access to healthcare services, and lack of proper education^20^.

In Africa, Various primary studies have reported a high prevalence of poor SCP among adult asthmatic patients. However, there is no study that estimates the overall level of SCP. Therefore, this systematic review and meta-analysis aimed to estimate the pooled prevalence of poor SCP among adult asthmatic patients in Africa, along with identifying associated factors.

The findings would hold significant implications for public health, which will provide information for the development of policies and strategies aimed at enhancing health monitoring for asthmatic patients. Additionally, policymakers can utilize these findings to allocate resources and plan effective care provisions to improve the level of SCP for these patients for better control of the diseases.

## Methods

### Reporting

This systematic review and meta-analysis was reported following the Preferred Reporting Items for Systematic Reviews and Meta-analyses (PRISMA) guidelines^21^. However, the study was not registered in the prospective registry for systematic reviews and meta-analyses (PROSPERO), as noted in the study’s limitations section.

### Databases and search strategy

To identify all the eligible literature, a systematic search from different electronic databases was conducted by five authors (AG, MS, TDK, HA, and YDM). The searched databases include Web of Science, CINHAL, PubMed/MEDLINE, Science Direct, Google Scholar, and university repositories. Searching was conducted using the following keywords: "Self Care, Self-Management, Patient Compliance, Asthma, asthmatic patients, bronchial asthma, Adult, determinant, predictor, barrier, risk factor, factor, and the name of the countries, combined by using AND and OR Boolean operators. The CoCo Pop framework defined the study focus as follows: Condition: assessment of prevalence of SCP; Context: Africa; and Population: adult asthmatic patients.

The search period spanned from November 1 to 18, 2025. Studies reporting the prevalence of poor SCP and/or its associated factors among adult asthmatic patients in Africa were included in the final meta-analysis. All retrieved articles were exported into EndNote Reference Manager (V.7) to organize citations and remove duplicate articles.

### Eligibility criteria

#### Inclusion criteria

Studies were included if they were conducted among adult asthmatic patients in Africa and reported on the level of asthma SCP and/or associated factors.

#### Exclusion criteria

Studies were excluded if they were interventional in design, review articles, lacked full-text availability, or were assessed as being of low methodological quality.

#### Outcome of interest

The primary outcome was the pooled prevalence of poor SCP among adult asthma patients in Africa. It was measured using various validated tools, as detailed in Supplementary material 1. The secondary outcome narratively synthesized factors associated with asthma SCP. Only variables reported as statistically significant across primary studies were summarized.

### Data extraction

Data were screened and extracted independently by four authors (GG, AAA, AAE, and AAM) using a Microsoft Excel spreadsheet. Any disagreements among the investigators were resolved through discussion until a consensus was reached. The data extraction form captured the following information from each study: first author’s name, year of publication, country where the study was conducted, study design, sample size, prevalence of poor SCP, and determinant variables, along with their adjusted odds ratios (AORs).

### Quality assessment

In this study, the quality of the included articles was assessed using the Newcastle–Ottawa Quality Assessment Scale for cross-sectional studies^22^. The tool evaluates three main domains: Selection (representativeness of the sample, sample size justification, non-response rate, and validation of measurement tools), Comparability (control for potential confounding factors through design or statistical adjustment), and Outcome (assessment methods and appropriateness of statistical analyses). Studies were awarded points within each domain, and those scoring 9–10 points were considered very good quality, 7–8 good quality, 5–6 satisfactory studies, and 0–4 unsatisfactory studies. Four independent authors (AL, MG, MAA, and GTM) assessed the studies, and any disagreements were resolved by calculating the average score of all assessors.

### Data processing and analysis

The extracted data from the included studies were cleaned in Microsoft Excel and exported to STATA version 17 for analysis. Heterogeneity across studies was assessed using Cochrane’s I^2^ statistic with the corresponding *p* value^23^. Heterogeneity was observed (I^2^ = 99.98%, *p* < 0.001), and therefore, a random-effects meta-analysis model was employed to estimate the pooled prevalence of poor SCP. We applied Hartung-Knapp adjustment for conservative random-effects inference, and reported 95% prediction intervals to characterize expected variation in future studies. Sensitivity analysis was performed to determine the influence of individual studies on the pooled estimate. Publication bias was evaluated using Egger’s test^24^ and visually illustrated with a funnel plot. The other extracted data were organized and displayed in a table. Statistical significance was set at *p* < 0.05 with a 95% confidence interval.

## Results

### Selection of studies

**Fig. 1:**
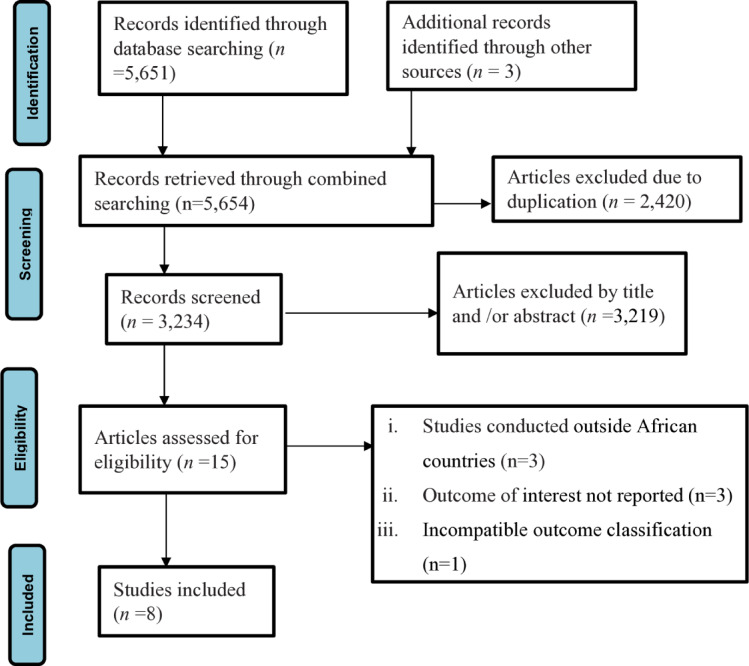
PRISMA 2020 flow diagram showing article selection process for systematic review of prevalence of poor self-care practice among adult asthmatic patients in Africa.

We collected 5654 articles from the different databases of PubMed/MEDLINE, Science Direct, Google Scholar, Web of Science, CINHAL, and university repositories. Then, 2420 duplicated records were removed before title and abstract review. Among the remaining 3234 studies, 3219 irrelevant studies were excluded after assessing and screening titles and abstracts. Then, 15 articles were assessed for eligibility. Of these, six studies were excluded: three studies^25–27^ were excluded for not reporting the outcome of interest, and an additional three studies^28–30^, were excluded due to being conducted outside African countries. One study^10^, was excluded due to incompatible outcome classification of asthma SCP. Ultimately, eight studies met the inclusion criteria and were included in this systematic review and meta-analysis (Fig. 1).

### Characteristics of included studies

This systematic review and meta-analysis incorporated nine cross-sectional studies, encompassing a total of 1859 participants, published within the period of 2020–2025. The sample sizes of the individual studies ranged from 68 to 470 participants. The included studies were reported from five countries: four studies were reported from Ethiopia^18,19,31,32^, with one study each contributed by Egypt^33^, Tunisia^34^, Kenya^35^, and Ghana^36^. In terms of statistical analyses, three studies^33,35,36^ employed the Chi-square (χ^2^) test, three studies utilized logistic regression^19,31,34^, and one study^18^ used linear regression to examine the associations between independent and outcome variables. Regarding the methods used to assess the level of SCP, only one study^33^ used an observational checklist; the remaining seven studies used interviews. In the quality assessment, four studies^19,31,33,34^ achieved a score of nine, two studies^35,36^ scored seven, and the remaining two studies were rated eight^18^ and six^32^. The most frequently encountered limitations pertained to sample representativeness, specifically concerning the sampling technique used for respondent selection. Nevertheless, the included studies generally exhibited robust study designs, employed validated measurement tools, and appropriately controlled for confounders, thereby ensuring the overall reliability of this review’s conclusions (Table 1).

**Table 1:** Characteristics of included articles for the prevalence of poor SCP among adult asthmatic patients in Africa.

First author’s name	Publication year	Country	Study design	Sample	*P* (%)	Methods of assessment	Quality
Yadesa et al. (2024)	2024	Ethiopia	CS	274	54.74	Interview	8
Abegaz et al. (2021)	2021	Ethiopia	CS	470	57.7	Interview	9
Geneti et al. (2025)	2025	Ethiopia	CS	413	48.4	Interview	9
Moyeta Bariso Gare et al. (2020)	2020	Ethiopia	CS	132	42.7	Interview	6
Elsadee et al. 2024	2024	Egypt	CS	100	72	Observation	9
Brobbey et al. (2025)	2025	Ghana	CS	68	58.8	Interview	7
Kagwaini et al. (2020)	2020	Kenya	CS	132	41	Interview	7
Dhaou et al. (2025)	2025	Tunisia	CS	190	68.4	Interview	9

NB: CS = cross-sectional.

### Pooled prevalence of poor SCP among adult asthmatic patients in Africa

**Fig. 2:**
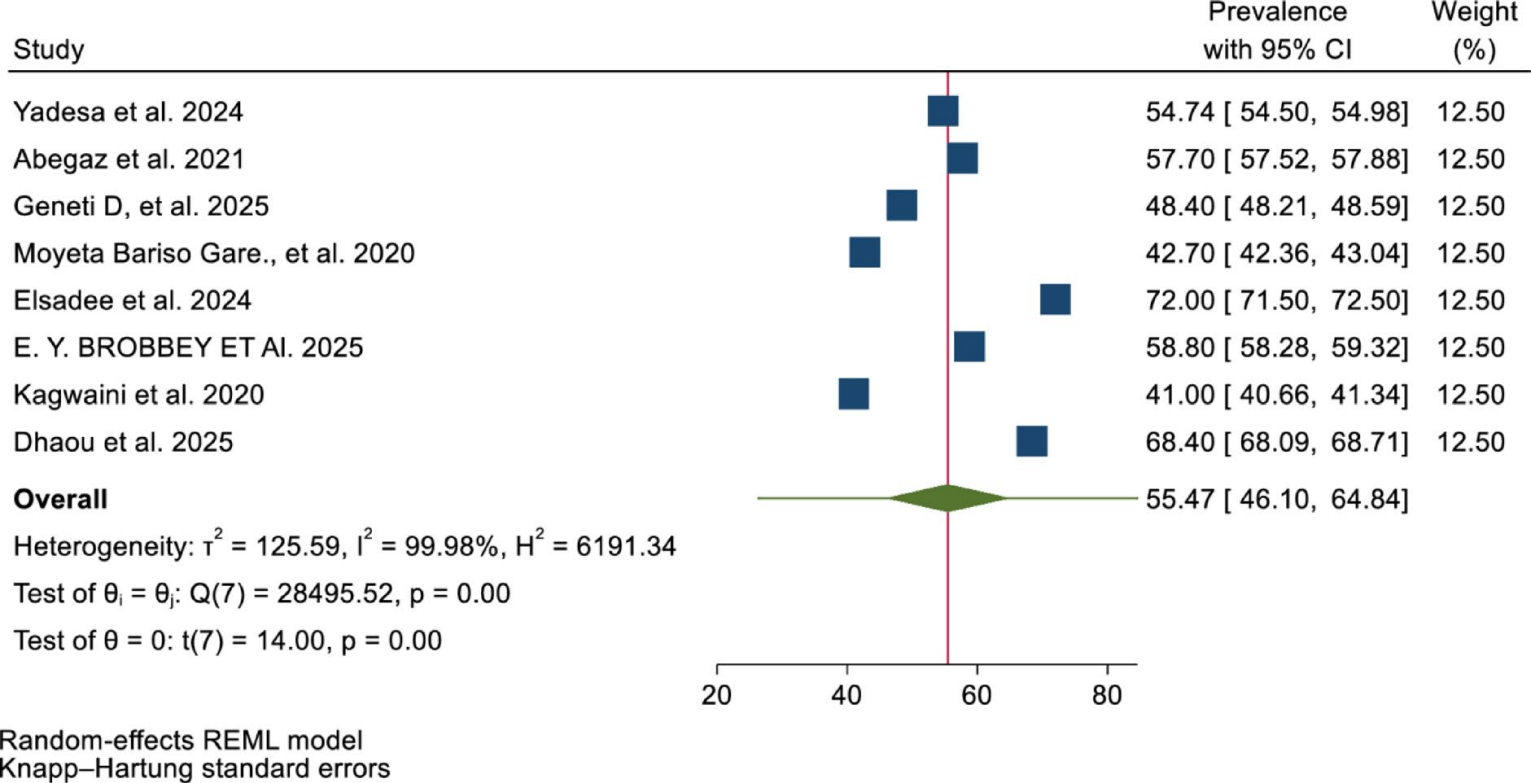
Forest plot of included studies that assess pooled prevalence of poor self-care practices among adult asthmatic patients in Africa.

In this systematic review and meta-analysis, the pooled prevalence of poor SCP was found to be 55.47% (95% CI = 46.10, 64.84) using the random-effects model. Significant heterogeneity was observed between studies, evidenced by an I^2^ test result of 99.98% (Fig. 2). Consequently, subgroup analyses were performed to identify potential sources of this high heterogeneity, based on geographic region (countries’ location), sample size, and scaling type. In the subgroup analysis by geographic region, the highest prevalence was reported in a study conducted in Northern Africa, with a prevalence of 70.19% (95% CI 47.32, 93.077%) and an I^2^ value of 99.31%.

The subgroup analysis based on sample size showed no statistically significant difference in pooled prevalence between the below average and above average subgroups (Tests of group differences *p* = 0.67), despite the highest prevalence being observed in the below average sample size subgroup (56.58% (95% CI 38.83, 74.33%)) and an I^2^ value of 99.98%. Similarly, the subgroup analysis based on scaling type showed no statistically significant difference in pooled prevalence between the Lickert scale and binary (yes/no) type subgroups (Tests of group differences *p* = 0.67), despite the highest prevalence being observed in the below average sample size subgroup (56.58% (95% CI 38.83, 74.33%)) and an I^2^ value of 99.98% (Table 2).

**Table 2:** Subgroup analysis results of the prevalence of poor SCP among adult asthmatic patients in Africa.

Subgroup analysis	Category	Number of articles	Prevalence With 95%CI	I^2^	*P* value
By sample size	Above average	3	53.61 (41.81, 65.42)	99.95%	0.003
	Below average	6	56.58 (38.83, 74.33)	99.98%	0.001
By geographical region	Horn of Africa	4	50.89 (40.23,61.54)	99.97%	0.001
	Northern Africa	2	70.19 (47.32, 93.07)	99.31%	0.016
	Other Sub-Saharan Africa	2	49.90 (40.67, 69.99)	99.97%	0.11
By scaling system	Lickert-scale	3	53.61 (41.81, 65.42)	99.95%	0.003
	Yes/no	6	56.58 (38.83, 74.33)	99.98%	0.001

### Meta regression

Meta-regression for the prevalence of poor SCP was conducted using sample size and year of publication as covariates. However, neither variable was found to be a significant source of heterogeneity (Table 3).

**Table 3:** Meta regression results of the prevalence of poor SCP among adult asthmatic patients in Africa.

No	Variables	Coefficient	Standard error	*P* value	95% CI
1	Year of publication	3.019	1.94	0.120	(− 0.79, 6.82)
2	Sample size	−0.014	0.03	0.624	(− 0.07, 0.04)

### Sensitivity analysis

A leave-one-out sensitivity analysis was conducted to assess the influence of individual studies on the pooled prevalence of poor SCP. The results showed that all point estimates remained within the overall 95% CI of the pooled prevalence, indicating that no single study had a disproportionate effect on the overall effect size (Table 4).

**Table 4:** Leave-one-out sensitivity analysis of the pooled prevalence of poor SCP among adult asthmatic patients in Africa.

No	Study omitted	Estimate (95% CI)
1	Yadesa et al. (2024)	55.57 (44.38, 66.76)
2	Abegaz et al. (2021)	55.15 (43.99, 66.31)
3	Geneti et al. (2025)	56.48 (45.65, 67.30)
4	Moyeta Bariso Gare et al. (2020)	57.29 (47.35, 67.23)
5	Elsadee et al. (2024)	53.11 (44.11, 62.10)
6	Brobbey et al. (2025)	54.99 (43.88, 66.10)
7	Kagwaini et al. (2020)	57.53 (47.98, 67.09)
8	Dhaou et al. (2025)	53.62 (43.71, 63.52)

### Publication bias

**Fig. 3:**
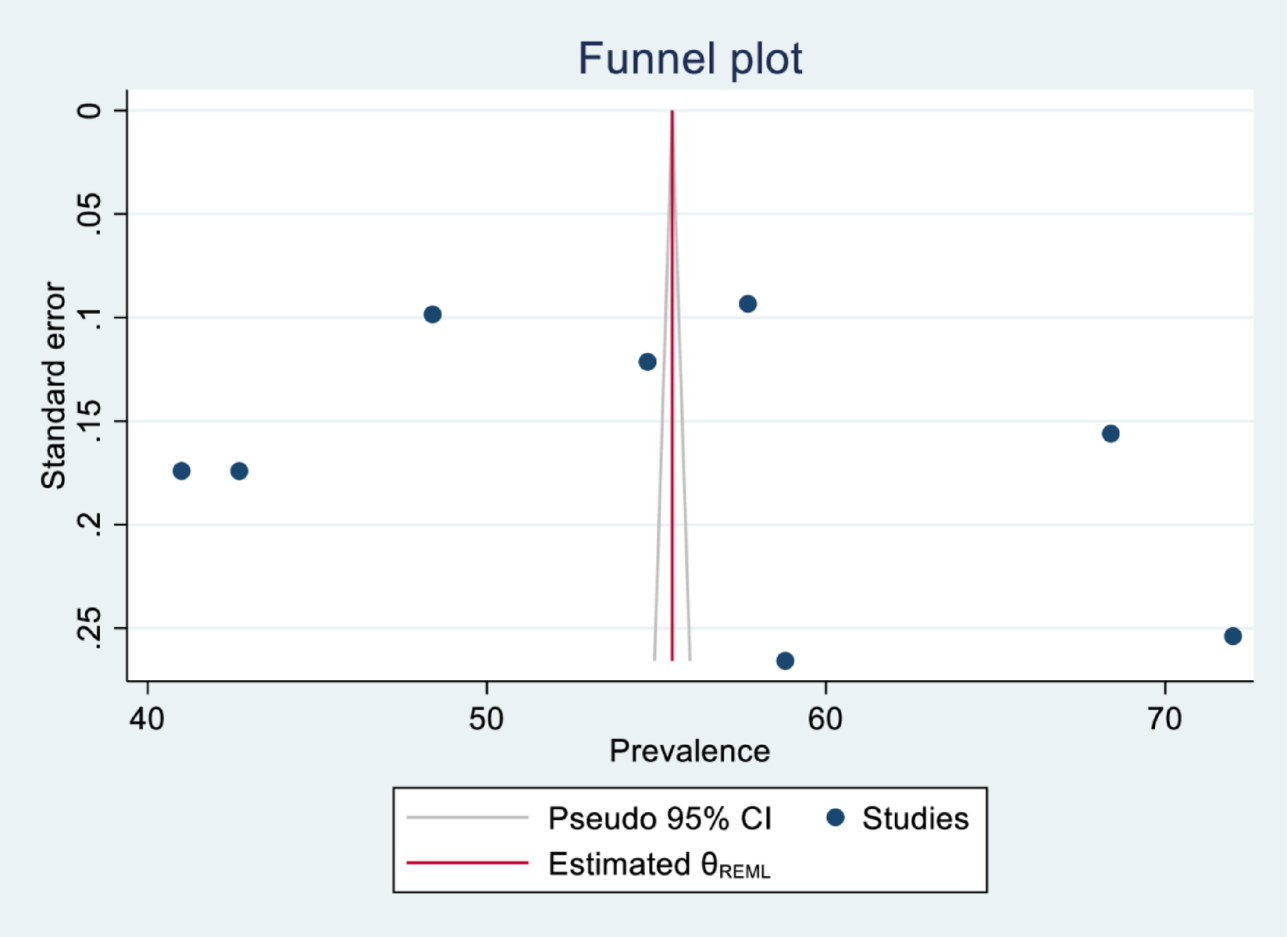
Funnel plot for assessing the presence of publication bias in the prevalence of poor self-care practice among adult asthmatic patients in Africa.

Publication bias was assessed using a funnel plot and Egger’s regression test. Egger’s test yielded *p* = 0.36, and the funnel plot appeared symmetrical (Fig. 3). Although these findings do not indicate significant publication bias, they should be interpreted cautiously due to the small number of studies and extreme heterogeneity (I^2^ = 99.98%), which limits the statistical power of these assessments.

### Factors associated with poor self-practice among adult asthmatic patients in Africa

Several socio-demographic, clinical, behavioral, and psychosocial factors were associated with asthma SCP.

### Socio-demographic related factors

#### Age

Age demonstrated significant associations with SCP across three studies, revealing a generally inverse relationship, where increasing age correlated with poorer SCP. As respondent age increased, SCP scores consistently declined. One study from Egypt found that patients aged 30–40 years had higher practice scores compared to those aged 60 and older (X^2^ = 12.26, *p* = 0.006)^33^. Similarly, a study from Ethiopia reported that individuals aged ≥ 55 years were 2.463 times more likely to exhibit poor SCP (AOR = 2.463, 95% CI 1.339–4.334, *p* < 0.05)^19^. Additionally, another Ethiopian study found that each additional year of age was associated with a 0.009-unit decrease in SCP (ß = −0.009, 95% CI −0.012–−0.005, *p* = 0.043)^18^.

#### Educational level

Educational level showed a significant association with SCP across two studies. In a study conducted in Kenya, patients with only primary education were significantly associated with poorer SCP (X^2^ = 24.954, *p* < 0.0001)^35^. Similarly, a study in Tunisia revealed that patients with only primary education were 2.774 times more likely to have poor SCP (OR = 2.774, 95% CI 1.436–5.360, *p* = 0.002)^34^.

#### Occupation

Occupation was significantly associated with SCP in one study conducted in Tunisia. The study found that retired individuals were eight times more likely to have poor SCP compared with those who were employed (OR = 8.143; 95% CI 2.404–27.584; *p* = 0.001)^34^.

#### Gender and marital status

Gender and marital status were identified as significant predictors of SCP in a study conducted in Tunisia. Female participants were less likely to exhibit poor self-management compared with males (OR = 0.533; 95% CI 0.285–0.995; *p* = 0.040). Furthermore, single individuals had significantly lower odds of poor SCP compared with married participants (OR = 0.254; 95% CI 0.110–0.520; *p* = 0.001)^34^.

### Clinical related factors

Comorbidity was identified as a significant predictor of SCP in two studies conducted in Ethiopia. Asthmatic patients with co-morbid illnesses were nearly two times more likely to have poor self-care practices compared with those without comorbid conditions (AOR = 1.860, 95% CI 1.063–3.254)^19^. Similarly, another study reported that participants without co-morbidities had higher odds of exhibiting good SCP compared with those with co-morbid conditions (AOR = 2.0, 95% CI 1.26–3.10; *p* = 0.03)^31^. Severity of asthma was also significantly associated with SCP in one study. The study revealed that patients with severe persistent asthma were 8.276 times more likely to have poor SCP compared with patients with intermittent asthma (OR = 8.276; *p* = 0.002)^34^.

### Behavioral and psychosocial related factors

#### Alcohol consumption

Alcohol consumption demonstrated significant associations with SCP across three studies, showing a generally inverse relationship between alcohol use and SCP. Participants who reported never drinking alcohol were 4.33 more likely to practice good self-care compared with those who had consumed alcohol (AOR = 4.33, 95% CI 2.52–7.44, *p* = 0.01)^31^. Similarly, individuals with a history of alcohol consumption were 1.832 more likely to exhibit poor self-care practices compared with non-drinkers (AOR = 1.832, 95% CI 1.136–2.954, *p* < 0.05)^19^. Furthermore, another study reported that alcohol drinking was negatively associated with SCP (β =  − 0.217, 95% CI − 0.338– − 0.082; *p* = 0.001), indicating that alcohol use decreased asthma SCP by 0.217^18^.

#### Smoking

Smoking demonstrated a significant association with SCP across four studies. Individuals who had never smoked were 6.67 times more likely to have good SCP compared with those who had smoked (AOR = 6.67, 95% CI 2.46–18.1; *p* = 0.01)^31^. Similarly, another study found that both passive smoking (OR = 9.482; 95% CI 1.235–72.793; *p* = 0.009) and former smoking (OR = 2.685; *p* = 0.017) were significantly associated with increased odds of poor SCP compared with non-smokers^34^. In addition, cigarette smoking was negatively associated with asthma SCP (β =  − 0.346, 95% CI − 0.529– − 0.180; *p* = 0.03), indicating that smoking decreased SCP scores by 0.346^18^. Furthermore, cigarette smoking was significantly associated with asthma SCP in another study (X^2^ = 12.44; *p* = 0.002)^35^.

#### Anxiety and depression

Anxiety demonstrated a significant association with asthma SCP across two studies. Individuals with borderline anxiety had higher odds of poor SCP compared with those without anxiety (AOR = 1.740; 95% CI 1.144–2.645)^19^. Similarly, Anxiety was negatively associated with SCP scores (β =  − 0.038; 95% CI − 0.060– − 0.019; *p* = 0.02), indicating that higher anxiety was associated with lower SCP. Depression was also negatively associated with SCP in one study (β =  − 0.029; 95% CI − 0.047– − 0.008; *p* = 0.005), showing that having depression decreased asthma SCP^35^.

#### Social support

Social support was significantly associated with asthma SCP across three studies. Individuals with social support were 1.915 more likely to have good SCP compared with those lacking support (AOR = 1.915; 95% CI 1.193–3.254)^31^. Similarly, asthmatic patients who had no social support were 1.57 times more likely to have poor self-care practice compared to those who had social support^19^. Furthermore, social support was positively associated with asthma self-management practice (β = 0.022; 95% CI 0.017–0.029; *p* < 0.001), indicating that for each one-unit increase in social support, asthma SCP increased by 0.022^35^.

## Discussion

The implementation of good SCP is essential for reducing symptoms, preventing exacerbations, lowering healthcare use, and ultimately improving the overall quality of life for asthmatic patients^37^. As per the investigators’ knowledge, this study represents the first systematic review and meta-analysis to comprehensively estimate the pooled level of poor SCP among adult asthmatic patients across Africa. Estimating this pooled prevalence holds significant public health implications, providing crucial data necessary for the development of targeted policies and strategies aimed at enhancing health monitoring and improving self-management outcomes for asthmatic patients.

This meta-analysis showed that the pooled prevalence of poor SCP among adult asthmatic patients in Africa, synthesized from eight studies with a total of 1779 participants, was found to be 55.47% (95% CI = 46.10, 64.84). This result suggests that more than half of the adult asthmatic population in Africa struggles with adequate self-care. This finding is largely consistent with similar studies conducted in Bangladesh (59.1%)^38^, and Taiwan (48.5%)^28^.

One of the most notable findings from this analysis was the high heterogeneity observed among the included studies, evidenced by an I^2^ value of 99.98% and a statistically significant *p* value = 0.00. This metric indicates substantial variability in the estimated pooled prevalence of poor SCP in adult asthmatic patients, which is likely attributable to differences in geographical regions and patient characteristics.

To address the observed variability, subgroup analyses were conducted based on geographic region (countries’ location), sample size and assessment tool scaling system. The analysis revealed significant differences in the prevalence of poor SCP across countries’ locations. The highest prevalence was reported in a study conducted in Northern Africa, with a prevalence of 70.19% (95% CI 47.32, 93.077%) and an I^2^ value of 99.31%. In contrast, the lowest prevalence was found in studies from the Horn of Africa, where the pooled prevalence was 50.89 (44.33, 57.45) and an I^2^ value of 99.97%. This difference might be due to cultural, socioeconomic, and health-system factors across countries^39^.

Subgroup analysis based on study sample size revealed no statistically significant difference in the pooled prevalence of poor SCP between the below-average and above-average subgroups (test for subgroup differences, *p* = 0.67). Specifically, the pooled prevalence for the below-average sample size group was 56.58% (95% CI 38.83%, 74.33%). While this was numerically higher than the above-average group, the difference did not reach statistical significance, suggesting that study population size does not significantly influence reported prevalence rates. Despite this lack of statistical variance between subgroups, the presence of extreme heterogeneity (I^2^ = 99.98%) implies that the observed variation in prevalence is almost entirely attributable to true differences between the studies rather than sampling error alone. Similarly, subgroup analysis based on assessment tool scaling (Likert scale vs. binary/yes–no types) showed no significant difference in pooled prevalence (*p* = 0.67). This consistency across different measurement formats suggests that the method of assessment much like the sample size is not a primary driver of the high prevalence of poor SCP observed in this meta-analysis.

This systematic review identifies multiple factors influencing asthma SCP, ranging from non-modifiable demographic factors to modifiable behavioral and psychosocial elements.

### Socio-demographic determinants

#### Age

Age emerged as a consistent predictor showing a clear inverse relationship with asthma SCP. Older patients, particularly those over 55 or 60, demonstrated significantly poorer SCP. The result is supported by several other studies done in USA^40^, Taiwan^28^, and United Arab Emirates^41^. This might be due to the fact that aging causes molecular and cellular damage that progressively impairs physical and mental capacities, heightening disease risk especially in older asthmatic patients who face forgetfulness and reduced attention to healthcare, hindering effective SCP^42,43^.

#### Educational level

Educational level significantly influences asthma SCP; patients with only primary education face substantially higher risks of poor SCP. This might be because lower education often limits health literacy, hindering comprehension of asthma management, medication adherence, and trigger recognition ultimately leading to poor SCP^44^.

#### Occupation

Occupation was also found to significantly influence asthma SCP, with retired individuals having higher odds of poor self-care practices compared to employed patients. This might be because retirement is associated with increased depression, life dissatisfaction, and hazardous drinking due to sudden life stressors such as reduced resources, weaker social support, smaller social networks, and the absence of workplace reminders or peer support for medication adherence and symptom monitoring, all of which undermine consistent SCP^45^.

#### Gender and marital status

Gender and marital status were identified as significant predictors of SCP. Female participants were less likely to exhibit poor SCP compared with males. This might be because women tend to have lower engagement in risky behaviors such as alcohol and tobacco use, greater health-seeking behavior and healthcare utilization, and stronger social support networks that provide emotional and practical support, thereby improving overall SCP^46,47^. Furthermore, single individuals had significantly lower odds of poor SCP compared with married participants. This might be due to the fact that, single individuals may dedicate more time to personal care, including exercise and adequate sleep, leading to higher physical activity levels, and they tend to be more autonomous and self-sufficient in managing their own health. Additionally, they often maintain broader social networks, engaging with friends, neighbors, and the community, which provides emotional and practical support that promotes the overall SCP^48^. 

### Clinical related factors

Comorbidity was identified as a significant predictor of poor SCP; asthmatic patients with comorbid illnesses were more likely to have poor SCP compared with those without comorbid conditions. This might be the fact that, co-morbidities can aggravate a patient’s health and make it more difficult to adhere to SCP, as managing multiple conditions can be overwhelming and physically or mentally taxing. Additionally, because symptoms of co-morbid conditions may resemble those of uncontrolled asthma, patients may struggle to distinguish which condition requires attention, leading to inconsistent or inappropriate self-care and increasing the risk of misdiagnosis, under-treatment, or over-treatment^49^. Similarly patients with severe persistent asthma were more likely to have poor SCP compared with patients with intermittent asthma. This might be due to the fact that severe asthma causes emotional distress like anxiety and depression, cognitive impairments affecting memory and medication adherence, physical fatigue limiting daily activities, reduced self-efficacy, competing comorbidities, and misunderstandings of condition severity that lead to underuse of preventives which collectively hinder effective asthma SCP^50^.

### Behavioral and psychosocial related factors

#### Alcohol consumption and Smoking

Alcohol consumption and smoking showed a significant inverse relationship with SCP, with individuals who consume alcohol and smoke being more likely to exhibit poorer SCP compared with non-drinkers and non-smokers. This might be due to the fact that, alcohol consumption and smoking can impair judgment, reduce motivation, and cause fatigue or anxiety, which decreases an individual’s ability to perform regular self-care activities. It also interferes with chronic disease management, promotes unhealthy lifestyle behaviors, and may be used as a coping mechanism for psychological stress, all of which contribute to poorer self-care practices^51–53^.

#### Anxiety and depression

Anxiety and depression are both significantly associated with poorer asthma self-care practice, with individuals experiencing these conditions more likely to exhibit reduced SCP. This result is supported by other studies^54,55^.

#### Social support

Social support was significantly associated with asthma SCP; individuals who had no social support were more likely to have poor SCP compared to those who had social support. This might be because support from family and friends is crucial for individuals with chronic illnesses, such as asthma, in several ways. Social support helps patients develop a positive self-concept and self-efficacy, providing the emotional and psychological foundation necessary for consistent SCP. Furthermore, social support enhances the practical implementation of asthma self-management, most notably by improving the extent of medication adherence and reinforcing healthy behaviors^56^.

### Limitations and strengths of the study

The main limitation of this systematic review and meta-analysis is the absence of PROSPERO registration, which was not undertaken because, at the time of the review’s inception, the registry did not allow the registration of systematic reviews focused on prevalence studies. The lack of uniform tools for assessing SCP and high heterogeneity were other limitations. Despite this, the overall findings remain robust, supported by several strengths: no individual study significantly influenced the pooled prevalence in the leave-one-out sensitivity analysis, and no publication bias was detected, although this should be interpreted cautiously due to the small number of studies and extreme heterogeneity. Importantly, this study represents the first attempt to synthesize evidence on the prevalence of poor SCP among adult asthmatic patients in Africa, providing crucial baseline data and highlighting the urgent need for further regional research to validate and refine these estimates.

## Conclusion and recommendations

In this systematic review and meta-analysis, the pooled prevalence of poor SCP among adult asthmatic patients across 8 African studies was 55.47% (CI 95% CI 6.10%, 64.84%), with extreme heterogeneity (I^2^ = 99.98%). The 95% prediction interval (26.38%, 84.55%) indicates substantial variation across settings. This summary statistic highlights the inconsistent burden of poor SCP and underscores the need for context-specific research and targeted interventions to improve asthma self-care in diverse African healthcare environments. Several factors were associated with poor asthma self-care practice, including socio-demographic factors (age, educational level, gender, marital status, occupation), clinical factors (comorbidity and asthma severity), behavioral factors (alcohol consumption and smoking), and psychosocial factors (depression, anxiety, and social support). Interventions to improve asthma self-care practice in Africa should adopt a holistic approach by targeting high-risk groups (such as older adults and those with low education or comorbidities), addressing modifiable behaviors like smoking and alcohol use, supporting mental health, and strengthening social support networks.

## Supplementary Information

Below is the link to the electronic supplementary material.

Supplementary Material 1

Supplementary Material 2

Supplementary Material 3

## Data Availability

The datasets used and/or analyzed during the current study are available from the corresponding author on reasonable request.

## Appendixes

Appendix A: Full search strings used for retrieve articles from PubMed.

## Abbreviations

AOR: Adjusted odds ratio
PROSPERO: Prospective registry for systematic reviews and meta-analyses
SCP: Self-care practice
