# Supplementary material for: Prevalence of poor self-care practice and associated factors among adult asthmatic patients in Africa: systematic review and meta-analysis
Source: Sci Rep. 2026 May 25;16:23917. doi: 10.1038/s41598-026-55252-y (PMC13433921; doi:10.1038/s41598-026-55252-y)
Supplement: Supplementary file 1 [file 41598_2026_55252_MOESM1_ESM.docx]

| Studies | Assessment tool | No of items | Scoring system | Cutoff point to define poor SCP |
| --- | --- | --- | --- | --- |
| Yadesa et al. 2024 | Asthma self-management questionnaire | 13 | Four-point Lickert scale | Below the mean |
| Abegaz et al. 2021 | Asthmatic  Self-care practice questionnaire | 8 | Four-point Lickert scale | Below the mean |
| Geneti D, et al. 2025 | Asthmatic  Self-care practice questionnaire | 8 | Four-point Lickert scale | Below the mean |
| Moyeta Bariso Gare., *et al*. 2020 | Asthmatic  Self-care practice questionnaire | 10 | Yes/no | Below the mean |
| Elsadee et al. 2024 | Asthma self-care practice assessment checklist | 82 | Yes/no (0-82) | <70% |
| E. Y. BROBBEY ET Al. 2025 | Asthma self-management questionnaire | 9 | Yes/no (0-9) | <=4 |
| Kagwaini et al. 2020 | Not described | 9 | Yes/no (0-9) | Not clearly described |
| Dhaou et al. 2025 | Asthma self-management questionnaire | 16 | Yes/no (0-100) | < 50 |
