# Supplementary material for: Prevalence of poor self-care practice and associated factors among adult asthmatic patients in Africa: systematic review and meta-analysis
Source: Sci Rep. 2026 May 25;16:23917. doi: 10.1038/s41598-026-55252-y (PMC13433921; doi:10.1038/s41598-026-55252-y)
Supplement: Supplementary file 2 [file 41598_2026_55252_MOESM2_ESM.docx]

| **Reason for Exclusion** | **Number of Studies (n=5,654)** |
| --- | --- |
| Articles excluded due to duplication | n=2,420 |
| Articles excluded by title and /or abstract | *n* =3,219 |
| Studies conducted outside African countries | n=3 |
| Outcome of interest not reported | n=3 |
| Incompatible outcome classification (n=1) | n=1 |
| Total excluded | 5,646 |
